# Basal Insulin Dose in Adults with Type 1 Diabetes Mellitus on Insulin Pumps in Real-Life Clinical Practice: A Single-Center Experience

**DOI:** 10.1155/2018/1473160

**Published:** 2018-06-05

**Authors:** Bartłomiej Matejko, Aneta Kukułka, Beata Kieć-Wilk, Agnieszka Stąpór, Tomasz Klupa, Maciej T. Malecki

**Affiliations:** ^1^Department of Metabolic Diseases, Jagiellonian University Medical College, Kraków, Poland; ^2^University Hospital, Kraków, Poland; ^3^Faculty of Electrical Engineering, Automatics, Computer Science and Biomedical Engineering, AGH University of Science and Technology, Kraków, Poland

## Abstract

**Introduction:**

Basal insulin (BI) infusion in pump therapy of type 1 diabetes (T1DM) mimics physiological secretion during the night and between meals. The recommended percentage of the total BI to daily insulin dose (termed the %BI) ranges between 30 and 50%. We analyzed whether this recommendation was followed in adults with T1DM from a university center, and whether BI doses were linked with glycemic control.

**Materials and Methods:**

We included 260 consecutive patients with T1DM (159 women and 101 men) treated with continuous subcutaneous insulin infusion at the Department of Metabolic Diseases, Krakow, Poland. Data were downloaded from patients' pumps and collected from medical records. We analyzed the settings of BI and the association of %BI with HbA1c level. Linear regression was performed.

**Results:**

The mean age of T1DM individuals was 26.6 ± 8.2 years, BMI was 23.1 ± 3.0 kg/m^2^, T1DM duration was 13.3 ± 6.4 years, and HbA1c level was 7.4%. There were 69.6% (*n*=181) of T1DM patients with %BI in the recommended range. The T1DM duration and HbA1c level of patients with a %BI <30% (*n*=23) was 9.5 years and 6.4%, respectively; for a %BI of 30–50%, it was 13.2 years and 7.4%; and for a %BI >50% (*n*=56), it was 15.8 years and 7.8% (*p* < 0.001 for both three-group comparisons). Multiple regression identified %BI among independent predictors of the HbA1c level.

**Conclusion:**

In this real-life analysis, the recommendations concerning %BI dosing were not followed by almost one-third of adult T1DM patients. Low %BI was associated with better glycemic control; however, this requires further confirmation.

## 1. Introduction

Continuous subcutaneous insulin infusion (CSII) therapy by insulin pumps has become a widely used treatment in patients with type 1 diabetes mellitus (TIDM). CSII is currently the most physiological method of insulin delivery available [1]. This mode of insulin therapy includes two components: basal insulin (BI) infusion that mimics physiological hormone secretion during the night and between meals, and boluses of insulin substituting acute postprandial insulin secretion [2]. The total BI dose is recommended to constitute 30–50% of the total daily insulin dose (DID) in all age groups of individuals with T1DM (the percentage of the total BI to daily insulin dose is termed here the %BI) [3–5]. One algorithm that is commonly used in adults with T1DM specifically suggests programming of BI as a half of the DID [6]. However, whether these recommendations are followed in real-life clinical practice and if %BI in adults with T1DM treated with CSII affects glycemic control have not been assessed so far. This is particularly intriguing in light of the clinical observations that keeping the %BI below 40% and encouraging the patient to bolus more frequently might lead to improved glycemic control with less weight gain [7].

The requirement for basal insulin varies throughout the day in healthy individuals and patients with T1DM [8–10]. The highest demand was reported in the morning (4:00–8:00 a.m.). This is related to the increased secretion of hormones (such as glucagon, adrenaline, and cortisol) that decrease insulin sensitivity [10, 11]. Their secretion results in a rise of blood glucose level in the early morning in many patients with T1DM, termed the dawn phenomenon. Daily fluctuations of insulin sensitivity are additionally influenced by physical activity, meals, and stress [8]. Indeed, it is a common practice for the CSII initiation to use three to four different BI rates per day to respond to changes in insulin sensitivity [12].

The proper BI dosing is crucial for achieving glycemic control [1, 13]. Some studies suggest that BI rate variability might be associated with severe hypoglycemic episodes or even with chronic diabetic complications [8, 14]. Moreover, both daily basal insulin requirement and circadian profile have been shown to be age-specific in patients with T1DM [15]. In addition, lower BI infusion was associated with a decreased HbA1c level in pediatric patients with T1DM [1, 13], although there are currently only limited data confirming this observation in adult patients. Similarly, the diurnal BI rate profile also remains insufficiently defined in adult patients with T1DM.

In this study, we analyzed whether the %BI recommendation was followed in adult patients with T1DM from a single university diabetes center, and whether BI settings were linked with glycemic control. The specific aims of this observational, retrospective study were to assess BI infusion, its daily patterns, and the association of %BI with HbA1c level.

## 2. Materials and Methods

We included 260 consecutive adult patients with T1DM (159 women and 101 men) treated with CSII and remaining under diabetes care of the Department of Metabolic Diseases, a tertiary university reference center in Krakow, Poland. We excluded individuals with proliferative retinopathy, diabetic kidney disease stage III or higher, pregnant women, and those using other antidiabetic agents as adjunct diabetes therapy or steroids. BI profiles were determined and verified at each clinical visit by certified diabetologists. Basal profile from a day directly preceding the patient's visit was used for the %BI analysis. Data regarding BI profiles, daily basal insulin, DID, average glycemia from blood glucose measurements, and average blood glucose measurements per day from the last two weeks were downloaded from dedicated software (CareLink Professional and Accu-Chek 360). Other collected variables were gender, age, type of insulin analog, weight, body mass index (BMI), T1DM duration, years on CSII, and the HbA1c level, which were all retrieved from the medical record. For the purpose of further analysis, we divided the study group according to their achievement of the recommended glycemic goal: HbA1c equal to or below 7.0%, or HbA1c above 7.0%.

We fitted the univariate regression models to identify significant predictors of HbA1c level among seven independent clinical variables: %BI, gender, age, BMI, T1DM, years on CSII, and average blood glucose measurements per day. Covariates significantly associated with HbA1c in univariate analysis were included in the multivariate model. To determine the differences between two groups, Student's *t*-test or Mann–Whitney *U* test was used; the Shapiro–Wilk test was used for the assessment of normality. To determine the differences between three groups, the ANOVA or Kruskal–Wallis test was used accordingly. To assess if there was a significant association between two categorical variables, the chi-square test was used. Statistical analyses were made in *R* statistical software version 3.4.1. The reported values for the whole group are represented as mean ± standard deviation or the median and range.

## 3. Results

The T1DM study group included 159 women (61.2%) and 101 men (38.8%). The patients were using rapid-acting insulin analogues in their personal insulin pumps including lyspro (*n*=75; 47.3%), aspart (*n*=68; 42.7%), and glulisine (*n*=16; 10%). The study individuals were on average 26.6 ±8.2 years old, with a BMI of 23.1 ± 3.0 kg/m^2^, and a T1DM duration of 13.3 ± 6.4 years. There were 69.6% (*n*=181) of T1DM patients with their %BI in the recommended range of 30–50% (mean proportion was 42.1%), while 8.8% (*n*=23) of patients had their %BI below and 21.6% (*n*=56) were above the recommended range. The T1DM duration and HbA1c level of patients with a %BI <30% was 9.5 years and 6.4%, respectively; for those with a %BI of 30–50%, it was 13.2 years and 7.4%; and for a %BI >50%, it was 15.8 years and 7.8% (*p* < 0.001 for both comparisons between the three groups). The HbA1c level and T1DM duration for patients with a %BI <30% were significantly lower than the other groups (post hoc for HbA1c: versus %BI 30–50% *p*=0.002, versus %BI >50% *p* < 0.001; for T1DM duration: versus %BI 30–50% *p*=0.009, versus %BI >50% *p* < 0.001). There was no statistical difference between the three abovementioned groups with respect to gender, age, and BMI.

Patients with less than 30% of %BI were characterized with higher total insulin requirement per kg than those with ≥30%, 0.83 IU versus 0.7 IU/kg, respectively, *p*=0.02. No correlation was found between %BI and BMI (*p*=0.88).

Patients with T1DM with optimal glycemic control (HbA1c level ≤7%; *n*=119) were using on average 0.68 IU/kg/day and 39.4% of %BI, while individuals with HbA1c >7.0% (*n*=141) were using 0.73 IU/kg/day and 44.5% of BI (*p*=0.016 and *p* < 0.0000, resp.). The two subgroups additionally differed with respect to age, DID, average glycemia, and number of blood glucose measurements per day. Detailed descriptive characteristics of the study cohort are provided in Table 1.

**Figure 1:**
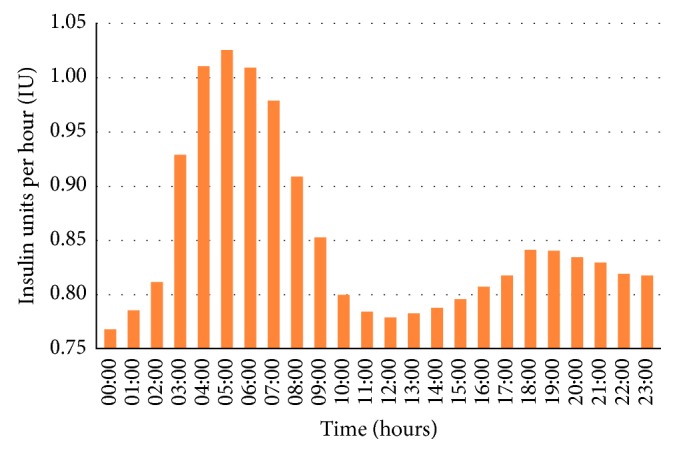
Circadian distribution of basal insulin in the whole study group.

The absolute values of BI in the circadian distribution are shown in Figure 1. We observed a bimodal pattern in the diurnal profile, with two phases of lower BI rates: one in the early part of the night (nadir of 0.77 IU/h between 0:00 and 1:00 a.m.) and one in the middle of the day (nadir of 0.78 IU/h between 12:00 and 1:00 p.m.). We also identified the largest peak of the insulin requirement between 5:00 and 6:00 a.m. (1.03 IU/h) and another smaller one between 6:00 and 7:00 p.m. (0.84 IU/h).

In the univariate analysis, an association between HbA1c level (as a dependent variable) and age, %BI, years on CSII, and average number of blood glucose measurements per day was found. In a multiple regression analysis with HbA1c level as a dependent variable, the same variables as mentioned above were independently correlated with HbA1c level (Table 2).

## 4. Discussion

In this observational study, we report that a substantial proportion of adults with T1DM remaining under the care of the university clinic use a %BI that is outside the recommended range. We also provide some evidence that lower than widely advised %BI might be associated with better glycemic control.

Choosing an optimal BI dose may help improve the effectiveness of therapy in patients with T1DM on insulin pumps. Most diabetes guidelines recommend that the BI rate should be programmed in hourly intervals, according to the patient's circadian variation of insulin requirement [16] and based on their individual fasting tests over a period of 6–10 h [14]. They also suggest that a %BI range of between 30% and 50% should be used [1–5, 15, 17]. We found that while this recommendation on %BI was followed in more than two-third of patients with T1DM, there was a substantial proportion of individuals using a %BI outside the recommended range.

Recent results from a Japanese population suggested that to achieve HbA1c <7.5%, the %BI should be set lower, to 30% [17]. This is also consistent with recent observational data from a pediatric population, where a drop of 10% in %BI resulted in a decrease of HbA1c by 0.22% across all %BI ranges [1]. These data are in-line with our results, as patients with T1DM using a %BI below 30% had their mean HbA1c lower than the rest of the participants.

Due to the nonrandomized and retrospective nature of this project, we cannot, however, claim that using a lower %BI would improve glycemic control in T1DM. Of note, the patients with a %BI below 30% had T1DM for a shorter period of time, which suggests that their residual insulin might have played a role in this association. However, the results of the multiple linear analysis indicated that %BI was an independent predictor of the HbA1c level. In fact, together with the number of daily glucose measurements and age of T1DM individuals, the %BI showed the strongest association with glycemic control. Therefore, in light of earlier reports and our study results, a question concerning a change of the current recommendation and lowering the advised range of %BI seems justified.

Of interest, we have shown that individuals with <30 %BI are characterized with higher total daily insulin requirement. We can speculate that it may be due to the need of delivering more correction boluses when being on less percentage of basal insulin or more precise meal bolus estimation.

Finally, our data on the circadian fluctuations of BI requirement generally confirmed earlier reports [8, 15, 18]. Therefore, four basal intervals with two peaks of the insulin requirement seem to be appropriate for adult patients with T1DM [12].

This report has some limitations. First, this was an observational study; thus, we cannot definitively prove any causative relationships, as a randomized, controlled study would be required for this purpose. Additionally, some important clinical variables that could potentially influence the results were not included in our analyses, for example, different types of boluses, omitted and additional boluses, and meal content, among others. Furthermore, we did not examine the residual insulin secretion capacity, for example, by measuring endogenous C-peptide [19]. Finally, data reported here were collected in a single reference diabetes clinic in Poland; thus, the conclusions of this study cannot be automatically extended to other centers.

## 5. Conclusion

In the real-life analysis from a diabetes university center, the recommendations concerning the %BI dosing were not followed by almost one-third of adult patients with T1DM on insulin pumps. Moreover, we identified some evidence for the association of low %BI with better glycemic control; however, this observation requires further confirmation in a larger, randomized, controlled study.

## Figures and Tables

**Table 1:** Study group characteristics and the comparison between T1DM patients with in-target HbA1c and above-target HbA1c are shown.

Variables	Whole group				HbA1c ≤7%		HbA1c >7%		*p*
	Mean	SD	Median	Range	Mean	SD	Mean	SD	
Age (years)	26.6	8.2	24.0	18–69	27.9	8.9	25.6	7.3	0.006
Weight (kg)	67.8	12.3	67.0	43–140	66.1	10.8	69.3	13.3	0.062
BMI (kg/m^2^)	23.1	3.0	22.6	16.7–40.9	22.8	2.8	23.3	3.1	0.213
T1DM duration (years)	13.3	6.4	13.0	2–37	13.2	6.7	13.5	6.2	0.479
Duration of CSII use (years)	6.8	3.6	7.0	1–17	6.4	3.7	7.1	3.5	0.091
HbA1c (%)	7.4	1.2	7.2	5.16–11.7	—	—	—	—	—
%BI (%)	42.1	10.1	42.0	14–78	39.4	9.7	44.5	10.0	0.000
DID (IU)	47.8	15.7	45.5	9–110	44.7	15.3	50.5	15.7	0.002
DID/kg (IU/kg)	0.71	0.20	0.69	0.11–1.62	0.68	0.21	0.73	0.19	0.016
Average glycemia from glucose meter (mg/dl)	157.3	34.0	155.0	88–279	143.4	28.8	169.2	33.7	0.000
Number of glucometer measurements per day (*n*)	5.4	3.0	5.0	0.2–15.5	6.1	3.2	4.7	2.6	0.010

BMI, body mass index; DID, daily insulin dose; CSII, continuous subcutaneous insulin infusion; %BI, percentage of basal insulin; IU, insulin units.

**Table 2:** Results of the univariate and multivariate linear regression analysis with HbA1c as the dependent variable.

Variable	Univariate analysis			Multivariate analysis	
	Estimate	95% CI	*p* value	Estimate	*p* value
%BI (%)	0.03	(0.01, 0.04)	<0.001	0.02	0.002
Gender (F)	0.14	(−0.14, 0.43)	0.327	—	—
Age at examination (years)	−0.03	(−0.04, −0.01)	0.001	−0.03	0.005
T1DM duration (years)	0.01	(−0.01, 0.03)	0.294	—	—
Duration of CSII (years)	0.05	(0.01, 0.09)	0.016	0.04	0.042
BMI (kg/m^2^)	0.03	(−0.02, 0.07)	0.24	—	—
Average number of blood glucose measurements per day (*n*)	−0.10	(−0.15, −0.05)	<0.001	−0.06	0.007

BMI, body mass index; CI, confidence interval; CSII, continuous glucose insulin infusion; %BI, percentage of basal insulin.

## Data Availability

The data used to support the findings of this study are available from the corresponding author upon request.
